# Hepcidin-to-Ferritin Ratio Is Decreased in Astrocytes With Extracellular Alpha-Synuclein and Iron Exposure

**DOI:** 10.3389/fncel.2020.00047

**Published:** 2020-03-10

**Authors:** Juntao Cui, Xinli Guo, Qijun Li, Ning Song, Junxia Xie

**Affiliations:** Institute of Brain Science and Disease, Shandong Provincial Key Laboratory of Pathogenesis and Prevention of Neurological Disorders, Shandong Provincial Collaborative Innovation Center for Neurodegenerative Disorders, Qingdao University, Qingdao, China

**Keywords:** neuroinflammation, ferritin, hepcidin, alpha-synuclein, primary astrocytes

## Abstract

Astrocytes are the most abundant glial cells in the central nervous system (CNS). As indispensable elements of the neurovascular unit, they are involved in the inflammatory response and disease-associated processes. Alpha-synuclein (α-syn) is released into the extracellular space by neurons and can be internalized by adjacent astrocytes, which activates glial cells to induce neuroinflammation. We were interested in whether astrocyte-mediated neuroinflammation is modulated by intracellular iron status and extracellular α-syn. Our results showed that recombinant α-syn (1 μg/ml and 5 μg/ml) treatment for 24 h did not affect the expression of the iron transporters divalent metal transporter 1 (DMT1) and ferroportin 1 (FPN1), nor those of iron regulatory protein (IRP) 1 or IRP2. Several proinflammatory cytokines, including tumor necrosis factor-α (TNF-α), interleukin (IL)-1β, and IL-6 exhibited up-regulated mRNA levels in 5 μg/ml α-syn-treated astrocytes. TNF-α release was increased, indicating that inflammatory responses were triggered in these cells. Pretreatment with the iron-overload reagent ferric ammonium citrate (FAC, 100 μmol/L) for 24 h had no effects on mRNA levels and release of proinflammatory cytokines. Inflammatory responses triggered by α-syn were not affected by iron overload. The iron chelator desferrioxamine (DFO, 100 μmol/L) exerted suppressive effects on TNF-α mRNA levels, although no change was observed for TNF-α release. Hepcidin mRNA levels were down-regulated significantly in astrocytes co-treated with FAC and α-syn, although independent treatment with either FAC or α-syn did not alter hepcidin levels. In contrast, hepcidin mRNA levels were up-regulated in DFO and α-syn co-treated cells. As expected, ferritin protein levels were up-regulated or down-regulated with FAC or DFO treatment, respectively. Following the up-regulation of ferritin mediated by α-syn, hepcidin-to-ferritin levels were indicative of modulatory effects in α-syn-treated astrocytes with altered iron status. Therefore, we propose that the hepcidin-to-ferritin ratio is indicative of a detrimental response in primary cultured astrocytes experiencing iron and extracellular α-syn.

## Introduction

Parkinson’s disease (PD) is characterized by a progressive loss of midbrain dopaminergic neurons in the substantia nigra (SN), resulting in motor symptoms including rigidity, postural instability, tremor at rest, and bradykinesia. A defining pathological feature of PD is the presence of intracellular protein aggregates termed Lewy bodies (LBs) in degenerated dopaminergic neurons, whose major components are aggregated alpha-synuclein (α-syn; Goedert, 2001; Dickson, 2012; Kalia and Lang, 2015; Przedborski, 2017). In addition to the presence of LBs in the most affected SN, α-syn aggregates have also been found in other brain regions, including the locus coeruleus, nucleus basalis of Meynert, hypothalamus, cerebral cortex, and cranial nerve motor nuclei, as well as in the central and peripheral divisions of the autonomic nervous system (Spillantini et al., 1997; Braak et al., 2010). Based on these findings, the prion hypothesis suggests that in the brain, α-syn released from degenerating neurons into extracellular spaces is taken up by neighboring neurons and non-neuronal cells (Volpicelli-Daley et al., 2011; Luk et al., 2012; Paumier et al., 2015; Brundin and Melki, 2017).

Astrocytes are the most abundant glial cells in the central nervous system (CNS). With widespread distribution throughout the whole CNS, astrocytes are responsible for extracellular homeostasis of water, ions, and neurotransmitters. Astrocytes act as indispensable elements of the neurovascular unit, which was an important participant in PD pathogenesis and treatment (Zou et al., 2017; Zhu et al., 2019). In a healthy brain as well as disease-associated processes, astrocytes were closely involved in inflammatory responses (Banks et al., 2015; Wang et al., 2018; Li et al., 2019). Neuroinflammation mediated by activated astrocytes/microglia were believed to be targeted to curb pathogenesis of neurodegeneration, or even promote the differentiation and proliferation of neural stem cells (Cho, 2019; Eremenko et al., 2019; Liu et al., 2019; Sn et al., 2019; Tu et al., 2019). Hepcidin, an antimicrobial peptide primarily secreted by the liver, is induced by inflammatory stimuli (Nicolas et al., 2002; Schmidt, 2015). In the brain, hepcidin is widely distributed in different brain areas and can be primarily induced in astrocytes by lipopolysaccharide (LPS) administration or by intracerebral hemorrhage (Xiong et al., 2016; You et al., 2017). Astrocytes have been reported to be able to internalize extracellular α-syn and activated to induce neuroinflammation (Lee et al., 2010, 2014). *In vitro* evidence showed that the co-culture of primary astrocytes with SH-SY5Y human neuroblastoma cells secreting α-syn resulted in the formation of astrocytic inclusion bodies. The induction of proinflammatory cytokines was correlated with the extent of intracellular accumulation of α-syn (Lee et al., 2010). More recently, amyloid-β (Aβ), another disease-associated misfolded protein was shown to induce inflammatory responses in astrocytes both *in vivo* and *in vitro* (Urrutia et al., 2013).

Iron is an essential trace element involved in various physiological processes, including oxygen transport (*via* hemoglobin), redox reactions, neurotransmitter synthesis, myelin production, and a number of mitochondrial functions. However, due to its propensity to release electrons and produce reactive oxygen species (ROS), excessive iron accumulation enables the occurrence of oxidative stress and ferroptosis, therefore contributing to the vulnerability of dopaminergic neurons in PD (Moreau et al., 2018; Trist et al., 2019). Astrocytes show robust expression of iron metabolism-related proteins. They set up the relative independence of iron balance in the brain by constituting the blood-brain barrier (BBB), and in addition, modulate synaptic activities by buffering iron concentration in the synaptic environment (Codazzi et al., 2015; Song et al., 2018). Under pathological conditions, astrocytes have the capacity to efficiently transport/recycle iron, thus potentially buffering excess iron and playing a crucial role in proper iron handling within the CNS (Pelizzoni et al., 2013; Zhang et al., 2013; Zarruk et al., 2015). As a regulator of brain iron homeostasis, hepcidin plays a key role in controlling the transport of iron across the BBB (Du et al., 2011; Urrutia et al., 2013). When treated with hepcidin peptide or infected with hepcidin expression adenovirus, astrocytes showed a significant capacity to reduce iron uptake and release (Du et al., 2011). There were controversial data that astrocytic hepcidin participated in LPS-induced neuronal apoptosis (You et al., 2017), or attenuate Aβ-induced inflammatory and pro-oxidant responses (Urrutia et al., 2017). However, the mechanisms by which astrocytic hepcidin responds to both altered iron metabolism and neuroinflammation has not been elucidated.

In the present study, we were interested in whether astrocyte-mediated neuroinflammation is modulated by intracellular iron status and extracellular α-syn. We demonstrated that extracellular α-syn did not affect iron metabolism-related proteins. Iron manipulations have limited effects on inflammatory response in primary cultured astrocytes triggered by α-syn. However, decreased hepcidin levels and a decreased ratio of hepcidin-to-ferritin were noted in astrocytes with co-administration of α-syn and iron. We further propose that the hepcidin-to-ferritin ratio could be indicative of detrimental interaction between iron and α-syn in primary cultured astrocytes.

## Materials and Methods

### Pharmacological Agents and Antibodies

α-syn was purchased from rPeptide (Bogart, GA, USA). The antibodies for ferric ammonium citrate (FAC), deferoxamine mesylate salt (DFO), and ferroportin1 (FPN1) were from Sigma–Aldrich (St. Louis, MO, USA). Fetal bovine serum (FBS) was from Gibco (Grand Island, NY, USA), and DMEM/F12 was from Hyclone (Logan, Utah, USA). Divalent metal transporter 1 (DMT1) antibody was from OriGene (Rockville, MD, USA), antibodies against ferritin, iron regulatory protein (IRP) 1 and IRP2 antibody were from Abcam (Cambridge, UK). The beta-actin antibody was from Bioss (Beijing, China). The Rat tumor necrosis factor-α (TNF-α), interleukin (IL)-1β, IL-6 Duoset ELISA Kit was from R&D Systems (Minnesota, MN, USA). All other chemicals and reagents were of the highest grade available from local commercial sources.

### Cell Culture and Treatment

Animals were handled in accordance with the National Institutes of Health Guide for the Care and Use of Laboratory Animals and were approved by the Ethical Committee of the Medical College of Qingdao University. Neonatal Wistar rats were sacrificed by cervical dislocation within postnatal days 1–2, and their mesencephalons were harvested. Several superficial washings were performed with phosphate-buffered saline (PBS) containing 100 U/ml penicillin and 100 μg/ml streptomycin, in order to limit contamination. Superficial blood vessels were carefully extracted using dissection pliers, and the tissues were mechanically dissociated to yield single cells. After filtration through a 100 μm pore mesh, the cell suspension was centrifuged at 1,000 *g* for 5 min and resuspended in DMEM/F12 cell culture medium containing 10% FBS, 100 U/ml penicillin, and 100 μg/ml streptomycin. The cells were plated in tissue culturing flasks pre-coated with poly-D-lysine at a density of five mouse brains per 75 cm^2^, then cultured at 37°C in a 5% CO_2_ incubator. After incubation for 24 h, the medium was changed and non-adherent cells were removed. Next, the adherent cells were incubated in culture medium for 10 d, with a medium change every 3–4 days. When cells had grown to confluence (after 10–14 days), the culture flasks were shaken on a rotary shaker at 200 rpm for 18–20 h at 37°C to remove any loosely attached microglia and oligodendrocyte precursor cells. The attached, enriched astrocytes were subsequently detached using trypsin-EDTA and then subjected to the different treatments. Recombinant α-syn was dissolved in sterile deionized water as a storage solution (1 mg/ml) and diluted to the appropriate concentration. FAC or DFO was dissolved in cell culture medium and diluted to 100 μmol/L. Astrocytes were treated by incubation with either α-syn (1 μg/ml or 5 μg/ml; Lee et al., 2010; Yu et al., 2016), FAC or DFO for 24 h. Alternatively, FAC or DFO pretreatment was applied for 24 h, followed by treatment with culture medium or α-syn (5 μg/ml) for a further 24 h. Astrocytes as control were treated with culture medium.

### Western Blotting

The cell lysate was prepared in ice-cold radio immunoprecipitation assay (RIPA) lysis buffer with a protease inhibitor (CWBIO, Beijing, China). Protein samples (20 μg) were loaded and separated by 8% or 10% sodium dodecyl sulfate-polyacrylamide gel electrophoresis (SDS-PAGE) at 80 V for 30 min, followed by 120 V for 90 min. The proteins were subsequently transferred onto a polyvinylidene fluoride (PVDF) membrane and the transfer was performed at 300 mA for 120 min on cold ice. Membranes were blocked for 1 h with 5% skimmed milk in Tris-buffered saline-tween-20 (TBST), and incubated overnight with the following primary antibodies at 4°C: DMT1 (1:800); FPN1 (1:800); IRP1 (1:2,000); IRP2 (1:1,000); ferritin (1:1,000); and Beta-Actin (1:5,000). Membranes were washed with TBST and incubated with goat anti-rabbit IgG-horseradish peroxidase secondary antibodies (1:5,000; Absin, Shanghai, China) for 1 h at room temperature. The blots were stained with the Clarity Western enhanced chemiluminescence (ECL) substrate (Millipore Corp, Billerica, MA, USA), and target bands were visualized using a UVP BioDoc-It Imaging System (Upland CA, USA). The target bands were quantified using the ImageJ software (NIH Image, Bethesda, MD, USA), and the density of each band was normalized against Beta-Actin.

### Real-Time Quantitative PCR

Total RNA was extracted from primary astrocytes with Trizol reagent (Invitrogen, Pittsburgh, PA, USA). According to the manufacturer’s instructions, the first-strand cDNA was synthesized using a RevertAid First Strand cDNA Synthesis Kit (Thermo Scientific Fermentas, Pittsburgh, PA, USA). Quantitative PCR was performed using specific primers for TNF-α (F5′-TCAGTTCCATGGCCCAGAC-3′, R5′-GTTGTCTTTGAGATCCATGCCATT-3′), IL-1β (F5′-CCCTGAACTCAACTGTGAAATAGCA-3′, R5′-CCCAAGTCAAGGGCTTGGAA-3′), hepcidin (F5′-GCCTGAGCAGCACCACCTAT-3′, R5′-AGCATTTACAGCAGAAGATGCAGA-3′), and GAPDH (F5′-AAATGGTGAAGGTCGGTGTGAAC-3′, R5′-CAACAATCTCCACTTTGCCACTG-3′). Gene expression was expressed as the mRNA level, normalized to that of the standard housekeeping gene GAPDH. Relative levels of target mRNA expression were calculated using the 2^−ΔΔct^ method. At least three independent experiments were performed for each measurement.

### Enzyme-Linked Immunosorbent Assay (ELISA)

The medium was collected and centrifuged for 20 min at 1,000 *g* to remove the pellet. The levels of TNF-α, IL-1β, IL-6 protein in culture medium were determined using the Rat TNF-α, IL-1β, IL-6 Duoset ELISA Kit, according to the manufacturer’s instructions. The optical densities of the standards and samples were measured by subtracting the readings at 540 nm from the readings at 450 nm using a multifunctional microplate reader (SpectraMax M5, Molecular Devices, San Jose, CA, USA).

### Statistical Analysis

All data were analyzed using GraphPad Prism version 6.0 software (GraphPad Software Inc., San Diego, CA, USA) All data were expressed as the mean ± SEM. Statistical analyses were performed by comparing the means of different groups using one-way ANOVAs with Tukey’s multiple comparisons test, with *P* < 0.05 considered significant.

## Results

### Extracellular α-Syn Did Not Affect the Expression Levels of Iron Metabolism-Related Proteins in Primary Cultured Astrocytes

**Figure 1:**
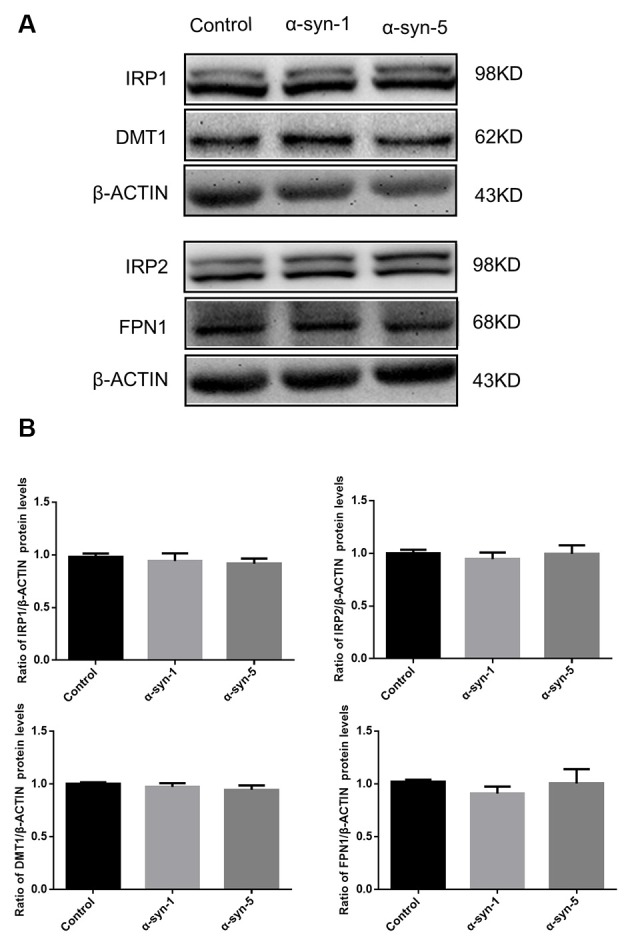
Changes in iron metabolism-related proteins in α-syn-treated primary cultured astrocytes. Proteins levels of DMT1, FPN1, IRP1 and IRP2 were unchanged **(A)**. Quantitative analysis of the proteins was shown in **(B)**. Data are presented as the ratio of DMT1, FPN1, IRP1 and IRP2 protein levels to β-actin. Each bar represents the mean ± SEM of three independent experiments.

We first investigated whether the expression levels of iron metabolism-related proteins in primary cultured astrocytes were affected by 1 μg/ml or 5 μg/ml α-syn treatment for 24 h. The protein levels of the iron importer DMT1, the iron exporter FPN1, and IRP1 and IRP2 were evaluated, as well as the mRNA levels of hepcidin. The results showed that there were no changes in any of these protein or hepcidin mRNA levels (Figures 1, 4), suggesting that neither 1 μg/ml nor 5 μg/ml α-syn had any effects on the iron transporting or iron regulatory components of astrocytes.

**Figure 2:**
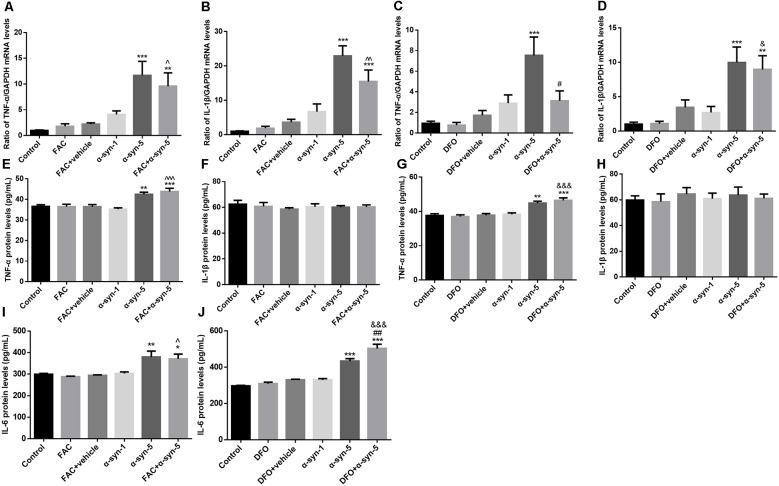
Effects of intracellular iron status and extracellularly administered α-syn on the mRNA levels of tumor necrosis factor-α (TNF-α) and IL1β and the release of TNF-α, IL1β, and IL-6 in primary cultured astrocytes. Treatment with 5 μg/ml α-syn induced a significant increase in TNF-α **(A,C)** and IL-1β **(B,D)** mRNA expression, as well as TNF-α **(E,G)** and IL-6 **(I,J)** release. DFO pretreatment suppressed the increase in TNF-α mRNA levels induced by 5 μg/ml α-syn **(C)** but enhanced IL-6 release **(J)**. IL-1β release is not affected by ferric ammonium citrate (FAC), DFO or α-syn **(F,H)**. Data are presented as the TNF-α and IL-1β mRNA levels relative to the level of GAPDH mRNA. Each bar represents the mean ± SEM of three independent experiments (*P < 0.05, **P < 0.01, and ***P < 0.001 compared with the control group; ^#^*P* < 0.05 and ^##^*P* < 0.01 compared with the α-syn-5 group; ^∧^*P* < 0.05, ^∧∧^*P* < 0.01, and ^∧∧∧^*P* < 0.001 compared with the FAC + vehicle group; ^&^*P* < 0.05 and ^&&&^*P* < 0.001 compared with the DFO+vehicle group).

**Figure 3:**
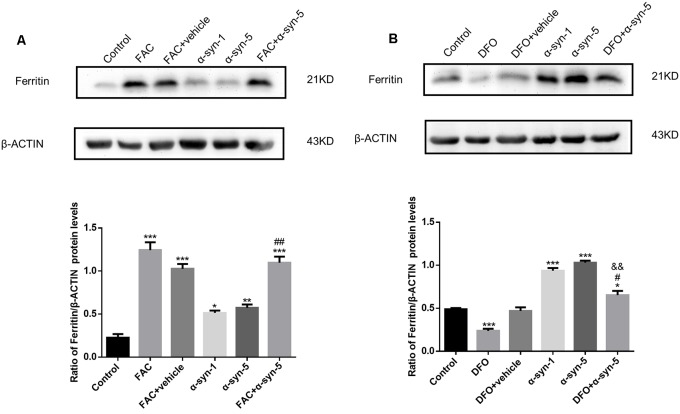
Effects of intracellular iron status and extracellularly administered α-syn on the protein levels of ferritin in primary cultured astrocytes. Both FAC and α-syn (1 μg/ml and 5 μg/ml) induced an up-regulation of ferritin protein levels. Ferritin protein levels were further increased in astrocytes co-treated with FAC and α-syn **(A)** and decreased in DFO/α-syn treated cells **(B)**, as compared to those in cells treated with α-syn treatment alone. Data are presented as the ratio of ferritin levels to β-actin. Each bar represents the mean ± SEM of three independent experiments (*P < 0.05, **P < 0.01 and ***P < 0.001 compared with the control group; ^#^*P* < 0.05 and ^##^*P* < 0.01 compared with the α-syn-5 group; ^&&^*P* < 0.01 compared with the DFO + vehicle group).

**Figure 4:**
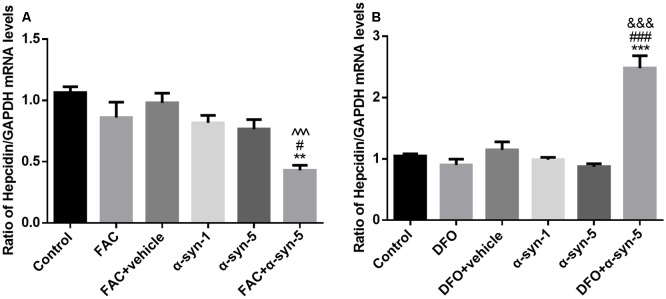
Effects of intracellular iron status and extracellularly administered α-syn on the mRNA levels of hepcidin in primary cultured astrocytes. Hepcidin mRNA levels were down-regulated significantly in astrocytes co-treated with FAC and α-syn **(A)** and up-regulated in DFO/α-syn treated cells **(B)**. Data are presented as the relative level of hepcidin mRNA to GAPDH mRNA. Each bar represents the mean ± SEM of three independent experiments (**P < 0.01 and ***P < 0.001 compared with the control group; ^#^*P* < 0.05 and ^###^*P* < 0.001 compared with the α-syn-5 group; ^∧∧∧^*P* < 0.001 compared with the FAC + vehicle group; ^&&&^*P* < 0.001 compared with the DFO + vehicle group).

### The Extracellular α-Syn Induced Inflammatory Response Was Largely Unaffected by Iron Status in Primary Cultured Astrocytes

Extracellular α-syn can be taken up by primary cultured astrocytes to trigger an inflammatory response (Lee et al., 2010). Therefore, we next investigated the effects of extracellular α-syn on TNF-α and IL-1β mRNA levels and on the release of the corresponding proteins by astrocytes. The results showed that treatment with 5 μg/ml, but not 1 μg/ml α-syn, induced a significant increase in TNF-α mRNA and IL-1β mRNA expression, indicating that inflammatory responses were triggered in these cells. Pretreatment with the iron-overload reagent FAC for 24 h had no effects on the mRNA levels of these proinflammatory cytokines, and also no effects on α-syn-triggered responses (Figures 2A,B). Release of TNF-α changed in parallel with the change in its mRNA expression; that is, TNF-α release was up-regulated in 5 μg/ml α-syn-treated astrocytes, with or without FAC pretreatment (Figure 2E).

Similar to FAC, the iron chelator DFO itself had no effects on the mRNA levels of these proinflammatory cytokines. DFO pretreatment exerted a suppressive effect on TNF-α mRNA levels, but not on IL-1β mRNA levels, induced by 5 μg/ml α-syn treatment (Figures 2C,D). However, no effects were observed on α-syn-induced TNF-α release (Figure 2G). Unfortunately, the release of IL-1β showed no change under any of these experimental manipulations (Figures 2F,H). More strikingly, we observed a stimulative effect of DFO on α-syn-induced IL-6 release, which was not observed in FAC-pretreated cells (Figures 2I,J). Overall, these results demonstrate that the extracellular α-syn-induced inflammatory response was not affected much by iron status in primary cultured astrocytes.

### Ferritin Protein Levels Were Tightly Regulated by Both Iron Status and by Extracellular α-Syn in Primary Cultured Astrocytes

We detected the expression of ferritin protein levels in astrocytes during extracellular α-syn treatment. We observed higher ferritin protein levels in α-syn-treated cells (1 μg/ml or 5 μg/ml; Figures 3A,B). As expected, robust regulation of ferritin expression was observed in FAC or DFO-treated cells; that is, ferritin protein levels were up-regulated or down-regulated by FAC or DFO treatment, respectively. Ferritin up-regulation induced by 5 μg/ml α-syn was enhanced by FAC pretreatment, whereas it was suppressed by DFO pretreatment.

### Hepcidin mRNA and Hepcidin-to-Ferritin Ratio Were Modulated by Iron Status in Primary Cultured Astrocytes Under Treatment of Extracellular α-Syn

Hepcidin is the key link between iron homeostasis and the regulation of acute inflammatory responses in the CNS (Raha et al., 2013; Vela, 2018). To explore the possible mechanisms involved in the modulatory effects of intracellular iron status and extracellular α-syn, we investigated the mRNA levels of hepcidin in primary astrocytes. As mentioned above, hepcidin mRNA levels were unchanged by either FAC or DFO treatment. Neither the 1 μg/ml nor the 5 μg/ml α-syn treatment had any effect on hepcidin, although there were increased mRNA or protein levels of proinflammatory cytokines, such as TNF-α, IL-1β, and IL-6. Unexpectedly, hepcidin mRNA levels were down-regulated significantly in astrocytes co-treated with FAC and α-syn (Figure 4A). In contrast, hepcidin mRNA levels were up-regulated in DFO and α-syn co-treated cells (Figure 4B), in parallel with an increase of IL-6 release in these cells (Figure 2J).

**Figure 5:**
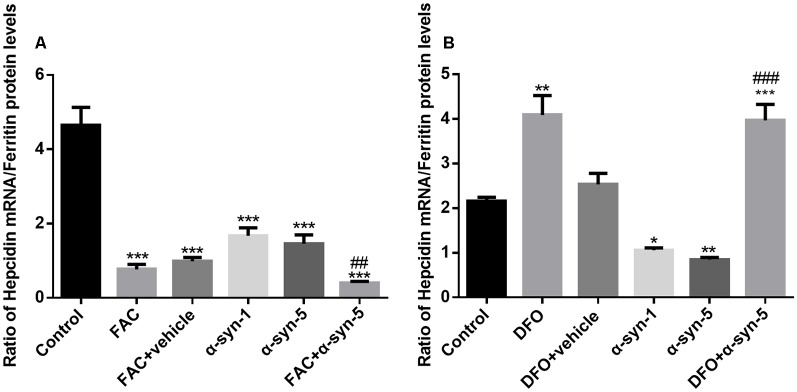
Effects of intracellular iron status and extracellularly administered α-syn on the ratio of hepcidin mRNA to ferritin protein levels in primary cultured astrocytes. The ratio of hepcidin mRNA to ferritin protein levels in astrocytes co-treated with FAC and α-syn was much lower than that in cells treated with α-syn alone **(A)**. However, the ratio was significantly higher in astrocytes co-treated with DFO and α-syn (**B**; *P < 0.05, **P < 0.01 and ***P < 0.001 compared with the control group; ^##^*P* < 0.01, ^###^*P* < 0.001 compared with the α-syn-5 group).

We then calculated the ratio of hepcidin mRNA levels to ferritin protein levels in astrocytes with altered iron status treated with extracellular α-syn. As shown in Figure 5A, the ratio of these two molecules in astrocytes with FAC and α-syn co-treatments differed from those with α-syn treatment alone, although there was no difference in the levels of proinflammatory cytokines (Figure 2). Similarly, this ratio was significantly higher in astrocytes during DFO and α-syn co-treatment (Figure 5B) and was therefore much more sensitive than the undetectable changes in proinflammatory cytokine levels.

## Discussion

Aggregation of α-syn is a central hallmark of neurodegeneration in PD and occurs extensively in the central and peripheral nervous systems (Cooper et al., 2018; Manfredsson et al., 2018). In recent years, cell-to-cell transfer of α-syn was believed to contribute to the progression of PD; that is, that α-syn may be released from neurons and then act on neighboring cells. Despite the fact that glial cells express low levels of α-syn, they are able to take up α-syn and thus contribute to the spread of α-syn pathology (Lee et al., 2010; Rey et al., 2016; Xia et al., 2019). As one of the functions of glial cells, neuroinflammation is a typical pathological trait characterizing various neurodegenerative diseases, including PD. Primary cultured astrocytes have higher uptake rate constants of extracellular α-syn compared to primary cultured neurons and microglia (Lee et al., 2008). Upon neuron-to-astrocyte transfer of α-syn, astrocytes display proinflammatory responses, producing multiple proinflammatory cytokines and chemokines (Fellner et al., 2013). *In vivo*, accumulation of α-syn in astrocytes, therefore an increased number of α-syn-positive astrocytes, is accompanied by an increase in proinflammatory cytokine levels in the Major Basic Protein (MBP)1-hα-syn transgenic mouse model of multiple system atrophy; these effects were reduced by antidepressant treatment (Valera et al., 2014). In the present study, we observed that 5 μg/ml α-syn induced a significant increase in TNF-α and IL-1β mRNA expression, as well as an increase of TNF-α and IL-6 release in primary astrocytes. Therefore, our results confirm that extracellular α-syn can cause an inflammatory response in primary cultured astrocytes.

Astrocytes are considered to be key regulators of the iron metabolism of the brain. The neurotoxin 6-hydroxydopamine (6-OHDA) induces iron deposits in primary cultured neurons but promotes iron transport rate in primary cultured astrocytes, suggesting distinct regulation of iron metabolism in neurons vs. astrocytes (Song et al., 2010; Zhang et al., 2013). Astrocytes have a high capacity for rapidly accumulating iron ions and various iron-containing compounds; they are able to store the iron in ferritin, although they do not normally contain large amounts of iron or ferritin (Bartzokis et al., 2007; Dringen et al., 2007). Ferritin sequesters iron in a non-toxic form, while the levels of free iron regulate cellular ferritin levels. Therefore, cytoplasmic ferritin synthesis is stimulated by an increase of iron, whereas it is decreased by iron depletion (Torti and Torti, 2002). When astrocytes are exposed to large concentrations of iron *in vitro*, their iron uptake rate temporarily exceeds the storage capacity of their ferritin and the cells experience transient oxidative stress (Hoepken et al., 2004). In the present study, cell iron status almost had no effect on the mRNA levels and release of pro-inflammatory factors in α-syn-treated primary astrocytes, although there seemed to be a suppression of TNF-α mRNA expression by DFO. We observed that the protein levels of the iron importer DMT1 and the iron exporter FPN1 were unchanged, indicating that extracellular α-syn does not affect iron transport in astrocytes. Ferritin protein levels were up-regulated or down-regulated with FAC or DFO treatment, respectively. We suppose that ferritin might efficiently buffer the intracellular free iron in astrocytes; thus, a negligible modulation of the α-syn-induced pro-inflammatory response was observed when iron levels were altered. Notably, ferritin levels were up-regulated in both the 1 μg/ml and 5 μg/ml in α-syn treated astrocytes, although IRP1 and IRP2 protein levels were unchanged. Ferritin responds to both inflammation and iron metabolism. Elevated ferritin in α-syn treated astrocytes may be due to the enhanced cytokine levels accompanying inflammation, considering that marked ferritin synthesis occurs at the post-transcriptional level during inflammation (Rogers et al., 1994; Thomsen et al., 2015).

Hepcidin is a key regulator of systemic iron homeostasis and modulates immune function in part by its ability to decrease iron absorption and serum iron content in response to infection and inflammation (Urrutia et al., 2017). Hepcidin acts on FPN1, the only iron efflux channel, inducing its internalization and degradation, eventually leading to iron retention in cells (Nemeth et al., 2004b). In macrophages, elevated hepcidin levels favor iron retention in these cells to encounter increased iron intake, infection, and inflammation. Whereas reduced hepcidin levels are associated with iron deficiency, hypoxia, anemia and homozygous hemochromatosis (Nemeth et al., 2004a; Sullivan, 2007). In the brain, hepcidin is produced in response to inflammatory stimuli in astrocytes. By modulating the levels of iron transporters, such as FPN1 and transferrin receptor 1, hepcidin is believed to contribute to brain iron homeostasis (Du et al., 2011; Urrutia et al., 2013). Both hepcidin and FPN1 are significantly reduced in hippocampal lysates from Alzheimer’s disease brains, although the reasons are unclear, this probably results from an imbalance in iron metabolism (Raha et al., 2013; Urrutia et al., 2013). At the cellular level, hepcidin promoter activity is significantly increased by the occupancy of c-Jun N-terminal kinase (JNK) or activator protein-1 (AP-1), which are enhanced through the direct activation of toll-like receptors (TLRs) induced by LPS (Lee et al., 2017). During infection, proinflammatory cytokines such as IL-6 promote transcriptional induction of hepcidin *via* signal transducer and activator of transcription (STAT) signaling (Hood and Skaar, 2012; Fillebeen et al., 2018). In the present study, hepcidin mRNA levels were unchanged under conditions of either altered iron levels (FAC/DFO treatment) or α-syn treatment. However, hepcidin mRNA levels were down-regulated significantly in astrocytes co-treated with FAC and α-syn; while they were up-regulated in DFO and α-syn co-treated cells, in parallel with an increase of IL-6 release in these cells. As ferritin is up-regulated dramatically and consistently in FAC treated astrocytes with/without α-syn, that means ferritin is not such a good indicator of α-syn exposure in iron-overloaded astrocytes, although ferritin could be moderately up-regulated by α-syn itself. More strikingly, we observed the calculated hepcidin-to-ferritin ratio was decreased in astrocytes with co-administration of α-syn and iron. The co-existence of iron and α-syn shifts the high/normal-hepcidin to low-hepcidin levels; the low hepcidin-to-ferritin ratio is efficient to reflect the situation in overloaded astrocytes experiencing extracellular α-syn, facilitating the iron-releasing phenotype in these cells. This might be detrimental for neighboring neurons who were vulnerable to both elevated iron and spreading α-syn. In contrast, in astrocytes with low iron levels and extracellular α-syn exposure, increased hepcidin levels and high hepcidin-to-ferritin ratio might be beneficial because of the possible iron retention in astrocytes and actually prevent iron elevation in the local environment *via* the large buffering capacity of the astrocytes. Given the importance of hepcidin and ferritin on iron homeostasis, the hepcidin-to-ferritin ratio, reflecting the amount of hepcidin relative to ferritin, underscores their roles in determining transcellular iron distribution. The relationship between this ratio and iron-inflammation interactions has not been reported previously, our study suggested a low hepcidin-to-ferritin ratio indicates a more unbeneficial situation occurring under conditions of both iron overload and proinflammatory insult.

In summary, in this study, we showed that the extracellular α-syn-induced inflammatory response was largely unaffected by iron status. Ferritin protein levels were tightly regulated by both iron status and inflammation. We observed decreased hepcidin and hepcidin-to-ferritin ratio in astrocytes with co-administration of iron and α-syn, and we propose the hepcidin-to-ferritin ratio is indicative of a detrimental response in primary cultured astrocytes experiencing extracellular α-syn and iron overload.

## Data Availability Statement

The datasets generated for this study are available on request to the corresponding author.

## Ethics Statement

The animal study was reviewed and approved by Ethical Committee of the Medical College of Qingdao University.

## Author Contributions

JX and NS designed the research. JC, XG and QL performed the research. JC and QL analyzed the data. NS and JC wrote the article, and all authors had access to the study data, and have reviewed and approved the final manuscript.

## Conflict of Interest

The authors declare that the research was conducted in the absence of any commercial or financial relationships that could be construed as a potential conflict of interest.
